# Association of cellular HIV-1 DNA and virological success of antiretroviral treatment in HIV-infected sub-Saharan African adults

**DOI:** 10.1186/s12879-022-07082-2

**Published:** 2022-01-29

**Authors:** Desmorys Raoul Moh, Jean-Baptiste Ntakpé, Delphine Gabillard, Arlette Ahoubet Yayo-Emieme, Anani Badjé, Gérard M. Kouame, Toni Thomas d’Aquin, Christine Danel, Xavier Anglaret, Serge P. Eholié

**Affiliations:** 1 Unité Pédagogique de Dermatologie et Infectiologie, UFR Sciences Médicales, Abidjan, Côte d’Ivoire; 2 Programme PAC-CI, 18 BP 1954, Abidjan 18, Côte d’Ivoire; 3 Inserm 1219, Université de Bordeaux, Bordeaux, France; 4 Centre de Diagnostic et de Recherches sur le SIDA (CeDReS), CHU de Treichville, Abidjan, Côte d’Ivoire

**Keywords:** HIV-1 DNA, Therapeutic success, Test and treat, Africa

## Abstract

**Background:**

HIV-1 DNA persists in infected cells, forming viral reservoirs. Pre-antiretroviral treatment (ART) HIV-1 DNA load was reported to predict ART success in European severely immunocompromised patients. The aim of this study was to determine whether HIV-1 DNA levels are associated with virological success in less severely immunocompromised patients who receive early ART in sub-Saharan Africa.

**Methods:**

The association between pre-ART HIV-1 DNA and the virological response after 30 months on ART was studied in multivariate logistic regression in patients randomised to immediate ART groups in the Temprano trial, which assessed the benefits of early ART in HIV-infected adults in Côte d’Ivoire. HIV-1 DNA was quantified in peripheral blood mononuclear cell (PBMC) using real-time PCR.

**Results:**

HIV-1 DNA levels were measured in 1013 patients. Their medians [IQR] of pre-ART CD4 count, HIV-1 RNA and HIV-1 DNA levels were 465 [379–578]/mm^3^, 4.7 [4.0–5.3] log_10_ copies/ml and 2.9 [2.5–3.2] log_10_ copies/million PBMC, respectively. Pre-ART HIV-1 DNA was significantly correlated with pre-ART HIV-1 RNA (R = 0.59, p < 0.0001). In multivariate analysis, HIV-1 DNA < 3 log_10_ copies/million PBMC was significantly associated with virological success at M30 after adjustment for other key variables (ART regimen, IPT, sex, age, WHO clinical stage, CD4 and HIV-1 RNA; aOR 1.57; 95% CI 1.08–2.30; p = 0.02).

**Conclusion:**

Low HIV-1 DNA was statistically associated with virological success in this population of sub-Saharan African adults who started treatment with a median pre-ART CD4 count at 465/mm^3^. HIV-1 DNA could become a useful tool for guiding some therapeutic decisions in the test-and-treat era.

*Trial registration* TEMPRANO ANRS 12136 ClinicalTrials.gov, number NCT00495651, date of registration 03/07/2007.

**Supplementary Information:**

The online version contains supplementary material available at 10.1186/s12879-022-07082-2.

## Introduction

It has been widely demonstrated that HIV-1 related morbidity and mortality rates have declined dramatically since antiretroviral treatment (ART) was introduced. It is notable that highly active ART reduces HIV-1 viral load and limits viral heterogeneity, and this has improved the life expectancy of people living with HIV-1 [1–3]. Although the extremely powerful and active combined ART renders the virus undetectable in the plasma, it cannot be totally eradicated from the body because it persists in the infected cells [4–6]. The size of this viral reservoir may be quantified approximately as the total HIV-1 DNA level in Peripheral Blood Mononuclear Cells (PBMC). The natural history of HIV-1 shows that this viral reservoir is established at an early stage, which then influences the course of the disease [7]. Disease progression, therapeutic success in people on ART and virological rebound in subjects whose treatment has failed are associated with high HIV-1 DNA levels, and are independent of the CD4 count and level of HIV-1 RNA in the plasma, as described by several previous studies [8–11]. These studies, however, were mainly carried out in industrialised countries in severely immunocompromised patients. Their sample sizes were relatively small and the studies took place prior to the WHO recommendation that all HIV-positive people should be treated regardless of their clinical, immunological or virological status. In HIV-1 patients starting ART regardless of CD4 count, there is little data on the value of HIV-1 DNA for predicting virological outcome [12].

The Temprano trial, a randomised controlled trial which we carried out to evaluate the benefits and risks of early ART and isoniazid preventive therapy (IPT) in West African HIV-positive adults, took place between 2008 and 2015. The participants were monitored for 30 months and the primary endpoint was severe morbidity.

In the present study, we looked for an association between pre-ART HIV-1 DNA levels in PBMC and M30 virologic success in HIV-infected adults who were randomised to early ART groups in the Temprano trial [1].

## Materials and methods

### Temprano trial

Temprano was a 2 × 2 factorial RCT conducted in Côte d’Ivoire. The trial design and results have been reported previously [1]. To summarise: the inclusion criteria were HIV infection, age ≥ 18 years, CD4 count ≤ 800/mm^3^ and no criteria for starting ART according to the most recent WHO guidelines. Participants were randomised into one of four arms: Arm-1 (Deferred-ART), in which ART was deferred until the WHO criteria for starting ART were met; Arm-2 (Deferred-ART + IPT), in which ART was deferred and a 6-month IPT prescribed; Arm-3 (Immediate-ART), in which ART was started immediately; Arm-4 (Immediate ART + IPT), in which ART was started immediately and a 6-month IPT prescribed.

After randomisation, all participants had a series of blood tests, including CD4 count and plasma HIV-1 RNA. Plasma and whole blood samples were also frozen at − 80 °C. All participants were monitored for 30 months. The primary endpoint was severe morbidity, defined as a combination of all-cause deaths, AIDS diseases, non-AIDS malignancies and non-AIDS-invasive bacterial diseases. The main result of the Temprano trial at month 30 (M30) was that early ART and IPT both reduced severe morbidity significantly.

### HIV-1 DNA and RNA measurement

PBMC HIV-1 DNA levels were measured on whole blood samples frozen at baseline. The HIV-1 DNA real-time PCR was carried out in the virology laboratory at the Necker University Hospital in Paris. The technique was based on amplification of the Long Terminal Repeat (LTR) gene, well adapted to HIV-1 non-B subtypes, and reference to the 8E5 cell line containing one HIV copy per cell (HIV DNA cell, Biocentric, Bandol, France) [13]. The threshold for the technique was five copies of HIV-1 DNA per PCR well. The HIV-1 DNA load was expressed as the proportion of infected cells in the cells targeted. Results were expressed as the HIV-1 DNA log_10_ copy number per million PBMC.

Plasma HIV-1 RNA was measured semestrially in all participants, using a real-time polymerase chain reaction assay (Generic HIV Charge Virale, Biocentric; detectability threshold 100 copies per millilitre) [1, 7, 14] (Additional file 1).

### Trial drugs

The first-line ART regimen consisted preferably of tenofovir/emtricitabine (300 mg + 200 mg oad; Truvada®, Gilead Sciences, Inc.) plus efavirenz (600 mg tad; Stocrin®, Merck Sharp & Dohme Corp.) Patients with contraindications to efavirenz (patients dually infected with HIV-1 and HIV-2, women who did not use effective contraception or had a history of nevirapine monotherapy for pMTCT) received tenofovir/emtricitabine plus lopinavir–ritonavir (100 mg/400 mg tad), or tenofovir/emtricitabine plus zidovudine (300 mg tad). The latter regimen was abandoned in December 2008 due to increased side effects in the upper digestive tract [15].

### Statistical analysis

All Temprano participants randomised to the immediate ART strategy and having a baseline HIV1-DNA quantification were included in the analysis. Univariate logistic regression models were used to look for an association between virological success at M30 and the following variables: baseline PBMC HIV-1 DNA, baseline HIV-1 RNA, baseline WHO clinical stage, first-line ART regimen prescribed at baseline, IPT prescribed at baseline, sex, age, and baseline CD4 count. Virological success at month 30 was defined as HIV-1 RNA < 100 copies/mL. HIV-1 DNA, CD4 count (regardless of the univariate analysis results) and other variables with a p < 0.25 in univariate analysis were included in the multivariate model. The primary analysis included all patients with available plasma HIV-1 RNA measurement at M30. We then carried out a sensitivity analysis considering missing HIV-1 RNA measurement at M30 as failures. The interaction between variables was tested. Statistical analyses were carried out using SAS® 9.3 software.

## Results

### Baseline and follow-up characteristics

**Fig. 1:**
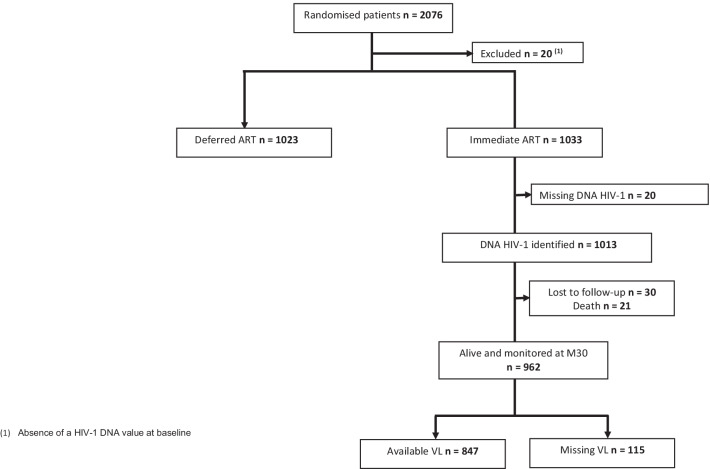
Flow chart: randomisation strategies

Between March 2008 and July 2012, 2076 patients were enrolled in the Temprano trial and randomly assigned to one of four treatment arms. 20 (1%) were subsequently excluded and 2056 (Immediate-ART n = 1033; Deferred-ART n = 1023) were included in the Temprano analysis at month 30. Of the 1033 patients included in the Immediate-ART arms, 20 (2%) were further excluded from the present analysis due to absence of a HIV-1 DNA value at baseline, and 1013 were included in the present analysis (Fig. 1).

The baseline and follow-up characteristics of the 1013 patients included in the analysis are shown in Table 1. At inclusion, 80% of our study population was female with a median age of 35 years (interquartile range [IQR] 30–42), 90% were asymptomatic or paucisymptomatic (WHO clinical stage 1 or 2). The medians (interquartile ranges [IQR]) of CD4 count, HIV-1 RNA and HIV-1 DNA levels were 465 [379–578]/mm^3^, 4.7 [4.0–5.3] log_10_ copies/ml and 2.9 [2.5–3.2] log_10_ copies/million PBMCs, respectively. The first-line ART regimen was TDF−3TC−EFV in 69%.

**Table 1:** Characteristics at inclusion and during follow-up (n = 1013)

*Inclusion*	
Age; years, median (IQR)	35 (30–42)
Sex; female, n (%)	808 (79.8)
WHO clinical stage 1 or 2; n (%)	912 (90)
CD4 cells/mm^3^	
Median (IQR)	465 (379–578)
Categories, n (%)	
< 350	197 (19.4)
350–499	392 (38.7)
≥ 500	424 (41.9)
Plasma HIV-1 RNA; log_10_ copies/ml*	
Median (IQR)	4.7 (4.0–5.3)
Categories; n (%)	
< 5	652 (64.6)
≥ 5	358 (35.4)
PBMC HIV-1 DNA; log_10_ copies/10^6^ cells	
Median (IQR)	2.9 (2.5–3.2)
Categories; n (%)	
< 3	576 (56.9)
≥ 3	437 (43.1)
1st line ART regimen; n (%)	
TDF/FTC plus EFV	700 (69.1)
TDF/FTC plus LPV/r	235 (23.2)
TDF/FTC plus AZT	78 (7.7)
*Follow-up*	
Duration of follow-up; patient years	2376
Lost to follow-up; n (%)	30 (3.0)
Death; n (%)	21 (2.1)
CD4 count at M30; cells/mm^3#^	
Median (IQR)	717 (548–887)
Categories; n (%)	
< 350	39 (4.6)
350–499	119 (14.0)
≥ 500	693 (81.4)
Plasma HIV-1 RNA at M30; log_10_ copies/ml^¤^	
Median (IQR)	0 (0–3.49)
Categories; n (%)	
< 100	711 (83.9)
100–999	29 (3.4)
1000–9999	42 (5.0)
10.000–99.999	32 (3.8)
≥ 100.000	33 (3.9)

*IQR* interquartile range; *WHO* World Health Organization; *ART* antiretroviral treatment; *TDF* tenofovir; *FTC* emtricitabine; *AZT* azidothymidine; *LPV/r* lopinavir/ritonavir; *DNA* desoxyribonucleic acid; *RNA* ribonucleic acid; *n* number of patients

*n = 1010 (3 missing values)

^¤^n = 847

^#^n = 851

The 1013 participants were monitored for a total of 2376 person years. 21 (2.1%) patients died and 30 (3%) were lost to follow-up.

**Fig. 2:**
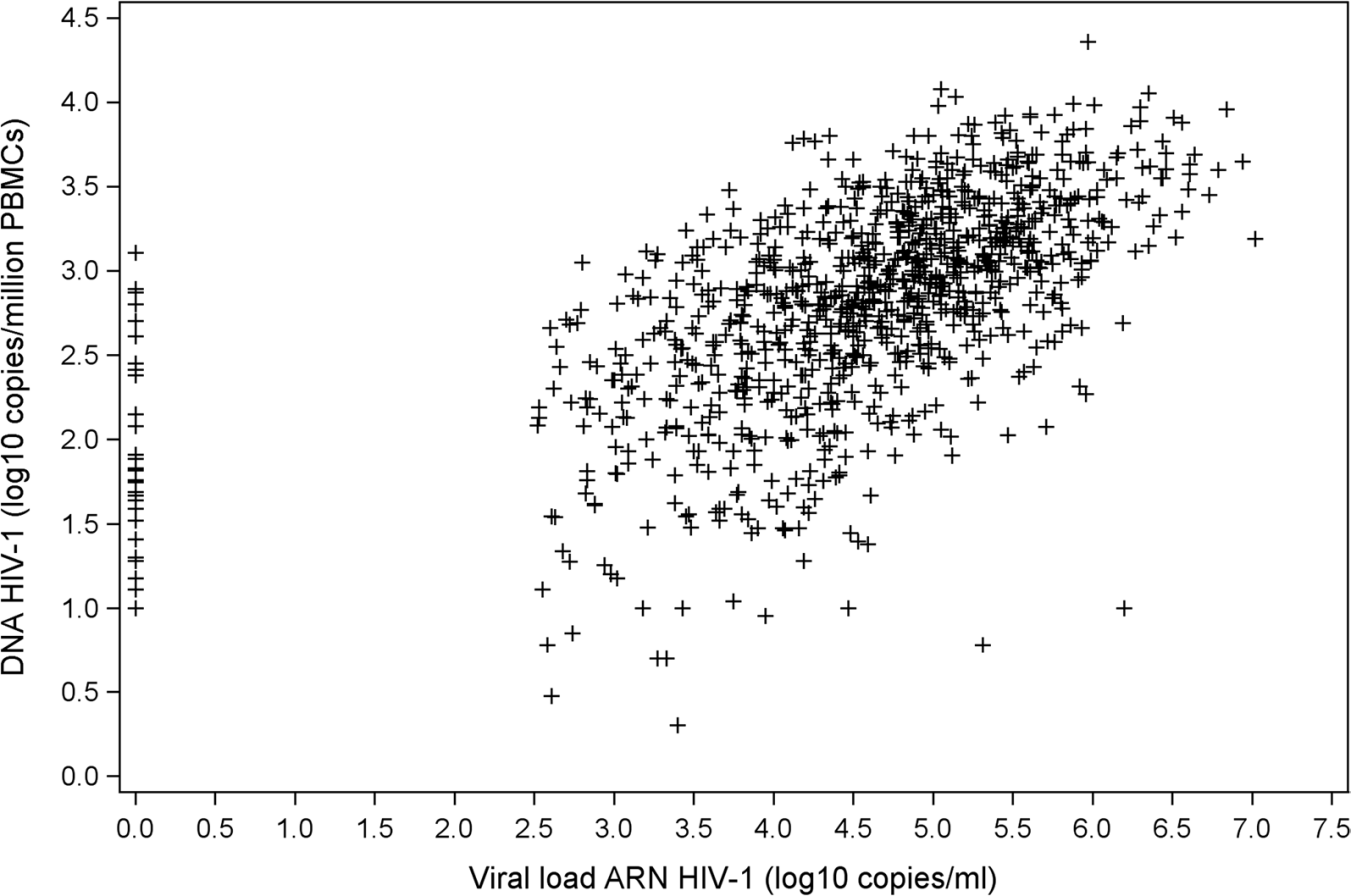
Correlation between HIV-1 DNA and HIV-1 RNA viral load (n = 1013)

Baseline HIV-1 RNA and HIV-1 DNA levels were significantly correlated (Spearman’s correlation: R = 0.59; *p* < 0.0001) (Fig. 2).

### HIV-1 DNA and M30 virologic success

At the end of the trial, 847/1013 (83.6%) patients had an HIV-1 RNA viral load available at M30, including 481/847 (57%) patients with baseline PBMC HIV-1 DNA < 3 and 366/847 (43%) with baseline PBMC HIV-1 DNA ≥ 3 log_10_ copies/10^6^ cells. 711 (83.9%) had achieved virologic success (VL undetectable < 100 copies/ml).

In the primary analysis (Table 2), HIV-1 DNA < 3 log_10_ copies/million PBMC was significantly associated with virological success at M30 (adjusted Odds Ratio 1.57; 95% CI 1.08–2.30; p = 0.02). There was no significant interaction between variables, including between CD4 count and HIV-1 DNA (p = 0.16).

**Table 2:** Factors associated with virological success at 30 months (n = 847)

	Univariate analysis			Multivariate analysis		
	OR	95% CI	p	aOR	95% CI	p
HIV-1 DNA: < 3 vs. ≥ 3 log_10_ copies/million PBMC	1.44	0.99–2.07	0.05	1.57	1.08–2.30	0.02
CD4 count: < 500 vs ≥ 500 cells/mm^3^	1.11	0.77–1.61	0.58	1.15	0.78–1.68	0.48
Plasma RNA: < 5 vs ≥ 5 log10 copies/ml	1.20	0.82–1.75	0.35			
Sex: male vs. female	1.32	0.82–2.15	0.25	1.15	0.69–1.91	0.60
Age: < 35 years vs. ≥ 35 years	0.60	0.42–0.87	0.01	0.62	0.42–0.91	0.01
WHO clinical stage: 1–2 vs. 3–4	0.76	0.39–1.47	0.41			
IPT: received vs. not received	1.00	0.69–1.44	0.98			
ART regimen						
TDF/FTC + LPV/r vs TDF/FTC + EFV	0.56	0.37–0.85	0.01	0.59	0.39–0.91	0.02
TDF/FTC + AZT vs TDF/FTC + EFV	1.28	0.59–2.77	0.53	1.41	0.64–3.11	0.40

*n* number of patients; *OR* odds ratio; *aOR* adjusted odds ratio; *IPT* 6-month isoniazide preventive therapy; *CI* confidence interval; *DNA* desoxyribonucleique acid; *PBMC* peripheral blood mononuclear cells; *RNA* ribonucleique acid; *LPV/r* lopinavir/ritonavir; *AZT* azidothymidine; *EFV* efavirenz; *TDF* tenofovir; *FTC* emtricitabine

For the missing-equal-failure sensitivity analysis (Table 3), there was a significant interaction between HIV-1 DNA and baseline CD4 count (p = 0.009). The analysis was therefore stratified for HIV-1 DNA and CD4 count. HIV-1 DNA < 3 log_10_ copies/million PBMC remained significantly associated with treatment success in patients with baseline CD4 < 500 cells/mm^3^ (aOR: 1.87, 95%CI 1.28–2.71, p = 0.001), but not in those with baseline CD4 ≥ 500 cells/mm^3^ (aOR 0.87, 95%CI 0.56–1.36, p = 0.55).

**Table 3:** Factors associated with virological success at 30 months

	Univariate analysis			Multivariate analysis		
	OR	95% IC	p	aOR	95% IC	p
HIV-1 DNA (< 3 vs ≥ 3 log_10_ c/MPBMC) amongst CD4 < 500/mm^3^	1.74	1.20–2.50	0.003	1.87	1.28–2.71	0.001
HIV-1 DNA (< 3 vs ≥ 3 log_10_ c/MPBMC) amongst CD4 ≥ 500/mm^3^	0.81	0.53–1.26	0.35	0.87	0.56–1.36	0.55
CD4 (< 500 vs ≥ 500/mm^3^) amongst HIV-1 DNA < 3 log_10_ c/MPBMC	1.83	1.26–2.64	0.001	1.79	1.23–2.61	0.002
CD4 (< 500 vs ≥ 500/mm^3^) amongst HIV-1 DNA ≥ 3 log_10_ c/MPBMC	0.86	0.56–1.32	0.48	0.84	0.54–1.30	0.43
HIV-1 RNA: < 5 vs ≥ 5 log10 copies/ml	1.11	0.84–1.46	0.48			
Sex: male vs. female	1.24	0.88–1.75	0.22	1.04	0.72–1.51	0.81
Age: < 35 vs. ≥ 35 years	0.62	0.47–0.81	0.0005	0.63	0.47–0.83	0.001
WHO clinical stage: 1–2 vs. 3–4	0.85	0.53–1.34	0.48			
IPT: received vs. not received	1.07	0.82–1.40	0.64			
ART regimen						
TDF/FTC + LPV/r vs TDF/FTC + EFV	0.61	0.44–0.83	0.002	0.63	0.45–0.87	0.005
TDF/FTC + AZT vs TDF/FTC + EFV	1.38	0.78–2.41	0.27	1.43	0.80–2.56	0.23

Sensitivity analysis (n = 1013)

*c/MPBMC* copies per million PBM; *n* number of patients; *OR* odds ratio; *aOR* adjusted odds ratio; *IPT* isoniazide preventive therapy; *CI* confidence interval; *DNA* desoxyribonucleic acid; *PBMC* peripheral blood mononuclear cells; *RNA* ribonucleique acid; *LPV/r* lopinavir/ritonavir; *AZT* azidothymidine; *EFV* efavirenz; *TDF* tenofovir; *FTC* emtricitabine

## Discussion

The objective of this study was to look for an association between PBMC HIV-1 DNA levels and virological success in HIV-infected West African adults enrolled in the Temprano trial and randomised to receive ART immediately regardless of the CD4 count.

We found that patients with low HIV-1 DNA level at baseline had a higher probability of virological success at 30 months than other patients, irrespective of plasma viral load and CD4 cell count.

Our results are obtained in a population with a low clinical stage and a relatively high median CD4 count. In the main analysis, the association between DNA and virological failure is significant, adjusted for CD4 count. In missing = failure, however, the interaction between CD4 and DNA led to stratification of the analyses. The association between DNA and the combination of virological failure, death or loss to follow up remained significant in people with less than 500 CD4, but was no longer significant in people with more than 500 CD4. This may illustrate the value of early ART. People who start ART with less than 500 CD4/mm^3^ have a higher risk of reaching this combined enpoint, and this risk is associated with the size of the reservoir. In people who start ART with a higher CD4 count, the reservoir may be less influential on the risk of virological failure, death or loss to follow up at 30 months.

Other studies previoulsy reported the association between HIV-1 DNA and virological success at 12–16 months of ART. These studies were conducted in Europe, and involved relatively low numbers of participants, having low CD4 counts [9, 12, 16–18]. This is the first time the association between HIV-1 DNA and virological success is reported in a population of sub-Saharan African adults who start ART with a high median pre-ART CD4 count.

HIV-1 DNA may provide complementary information to standard markers, CD4 count and plasma HIV-1 RNA [9]. In our study, baseline HIV-1 DNA and RNA levels were significantly correlated. The correlation coefficient, although slightly higher than in a previous study [12], was low. This suggests a different role for these two markers. HIV-1 RNA load reflects the degree of ongoing HIV-1 replication, while HIV-1 DNA reflects the level of the reservoir, including integrated and non-integrated viral genomes encoding competent and defective viruses.

As long as current treatments are unable to significantly decrease the reservoir level, HIV-1 DNA is not likely to be a useful tool for monitoring the efficacy of ART. However, it can be useful in guiding the choice of specific therapeutic strategies. In the MONARK therapeutic lightening trial, ART-naïve patients with low HIV-1 DNA had a higher rate of success on ritonavir-boosted lopinavir monotherapy compared with other patients, suggesting that simplified one-drug treatments may be safer in patients with low reservoir levels [19].

Our study has several limitations:

Firstly, the association of a marker with prognosis in a cohort study should be interpreted with caution, as there may be one or more confounding factors that are not accounted for in the analyses.

Secondly, HIV-1 DNA in peripheral blood mononuclear cells is a reflection of the reservoir, but do not represent the entire reservoir in view of its heterogeneity [20, 21]. However, HIV-1 DNA in peripheral blood has been shown to be highly correlated with HIV-1 DNA in other tissues (lymphoid tissue, intestinal mucosa, rectal) in several studies [14, 22].

## Conclusion

Cellular HIV-1 DNA is an interesting tool, complementary to plasma viral load. As a marker of the viral reservoir, it may be proposed as an efficacy endpoint in trials of therapies aimed at eradicating the virus in the future. In the present time, it may also help identify individuals at higher risk of virological failure, and thus allow for the adaptation of monitoring or the choice of treatments in these patients.

## Supplementary Information

**Additional file 1.** Methods for HIV-DNA quantification.

## Data Availability

The datasets generated and analyzed during the current study are not yet publicly available because the study sponsor’s datasharing procedures are currently under review, but they are available from the corresponding author on reasonable request.
