# Supplementary material for: Association of cellular HIV-1 DNA and virological success of antiretroviral treatment in HIV-infected sub-Saharan African adults
Source: BMC Infect Dis. 2022 Jan 29;22:100. doi: 10.1186/s12879-022-07082-2 (PMC8800335; doi:10.1186/s12879-022-07082-2)
Supplement: Supplementary file 1 — Additional file 1. Methods for HIV-DNA quantification. [file 12879_2022_7082_MOESM1_ESM.pdf]

# Additional file

Methods for HIV-DNA quantification

HIV-1 DNA quantification was performed using Generic HIV DNA Cell (Biocentric, Bandol, France). Primers sequence presented below were previously described (Avettand-Fenoel et al, J Med Virol 2009)

**Forward primer (NEC005)** : 5'-GCCTCAATAAAGCTTGCC-3'

**Reverse primer (NEC131)** : 5'-GGCGCCACTG-CTAGAGATTTT-3'

**TaqMan probe (MLC1) MGB LTR** : 5'-AAGTRGTGTGT-GCCC-3'

5' reporter, 6-carboxyfluorescein (FAM) and the 3' quencher, 6-carboxytetramethylrhodamin (TAMTRA) (Applied Biosystems, Foster City, CA).

Schvachsa et al. 2007 showed excellent correlation between the quantification of HIV-1 RNA and HIV-1 DNA of non-B subtypes using this primer and probe set.

The distribution of HIV-1 subtypes found in Côte d'Ivoire is presented in the table below (from Rouet F et al J Acquir Immune Def Syndr 2007)

**TABLE 1.** Group/Subtype Distribution of Collected Samples: Necker Hospital, Paris, France

| Genetic Group/Subtype | Panels | | Clinical Samples | Total (%) |
|---|---|---|---|---|
| | WHO | ANRS AC11 | | |
| Group M | | | | |
| B subtype | 1 | 0 | 266 | 267 (51.5) |
| Non-B subtypes | | | | |
| Subtype A | 1 | 11 | 30 | 42 (8.1) |
| Subtype C | 1 | 15 | 7 | 23 (4.4) |
| Subtype D | 1 | 8 | 10 | 19 (3.7) |
| Subtype F | 1 | 8 | 8 | 17 (3.3) |
| Subtype G | 1* | 9 | 9 | 19 (3.7) |
| Subtype H | 1 | 7 | 4 | 12 (2.3) |
| Subtype J | 0 | 2 | 2 | 4 (0.8) |
| CRF01_AE | 1 | 1 | 4 | 6 (1.2) |
| CRF02_AG | 0 | 3 | 60 | 63 (12.2) |
| CRF05_D/F | 0 | 0 | 1 | 1 (0.2) |
| CRF06_cpx | 0 | 7 | 8 | 15 (2.9) |
| CRF09_cpx | 0 | 0 | 1 | 1 (0.2) |
| CRF11_cpx | 0 | 4 | 2 | 6 (1.2) |
| CRF12_BF | 0 | 2 | 0 | 2 (0.4) |
| CRF14_BG | 0 | 1 | 0 | 1 (0.2) |
| Unclassified | 0 | 0 | 18 | 18 (3.5) |
| Total | 7 | 78 | 164 | 249 (48.1) |
| Group O | 1 | 0 | 0 | 1 (0.2) |
| Group N | 1 | 0 | 0 | 1 (0.2) |
| Total | 10 | 78 | 430 | 518 |

*AG-GH (RU570) strain.

## References

Schvachsa N, Turk G, Burgard M, Dilernia D, Carobene M, Pippo M, et al. Examination of real-time PCR for HIV-1 RNA and DNA quantitation in patients infected with HIV-1 BF intersubtype recombinant variants. J Virol Methods 2007;140:222-227

Rouet F, Chaix ML, Nerrienet E, Ngo-Giang-Huong N, Plantier JC, Burgard M, et al.. Impact of HIV-1 genetic diversity on plasma HIV-1 RNA Quantification: Usefulness of the Agence Nationale de Recherches sur le SIDA second-generation long terminal repeatbased real-time reverse transcriptase polymerase chain reaction test. J Acquir Immune Defic Syndr 2007; 45:380–388
